# TRPV4 Promotes Metastasis in Melanoma by Regulating Cell Motility through Cytoskeletal Rearrangement

**DOI:** 10.3390/ijms232315155

**Published:** 2022-12-02

**Authors:** Shuai Huang, Suyun Yu, Rui Deng, Huan Liu, Yushi Ding, Yifan Sun, Wenxing Chen, Aiyun Wang, Zhonghong Wei, Yin Lu

**Affiliations:** 1 Jiangsu Key Laboratory for Pharmacolgy and Safety Evaluation of Chinese Materia Medica, School of Pharmacy, Nanjing University of Chinese Medicine, Nanjing 210023, China; 2 School of Medicine & Holistic Integrative Medicine, Nanjing University of Chinese Medicine, Nanjing 210023, China; 3 Jiangsu Collaborative Innovation Center of Traditional Chinese Medicine (TCM) Prevention and Treatment of Tumor, Nanjing University of Chinese Medicine, Nanjing 210023, China

**Keywords:** TRPV4, Baicalin, calcium influx, metastasis, cofilin

## Abstract

The abnormal expression of Transient Receptor Potential cation channel subfamily V member 4 (TRPV4) is closely related to the progression of multiple tumors. In addition, TRPV4 is increasingly being considered a potential target for cancer therapy, especially in tumor metastasis prevention. However, the biological correlation between TRPV4 and tumor metastasis, as well as the specific role of TRPV4 in malignant melanoma metastasis, is poorly understood. In this study, we aimed to examine the role of TRPV4 in melanoma metastasis through experiments and clinical data analysis, and the underlying anticancer mechanism of Baicalin, a natural compound, and its inhibitory effect on TRPV4 with in vivo and in vitro experiments. Our findings suggested that TRPV4 promotes metastasis in melanoma by regulating cell motility via rearranging the cytoskeletal, and Baicalin can inhibit cancer metastasis, whose mechanisms reverse the recruitment of activated cofilin to leading-edge protrusion and the increasing phosphorylation level of cortactin, which is provoked by TRPV4 activation.

## 1. Introduction

Metastasis is the leading cause of cancer-related death and the treatment strategies have not substantially progressed in the last few years [1]. Metastasis is a multistage process, and several mechanistic pathways can mediate each of the requisite steps [2]. Although several treatment therapies for malignant tumors have been developed, the diagnosis of metastatic disease inevitably leads to terminal illness [3,4,5,6], especially metastatic melanoma, which carries a grim prognosis, with a median survival of 9 months and a long-term survival rate of 10% [7]. Thus, the development of potential therapeutic drugs for melanoma metastasis is necessary.

Ion channels are crucial for normal cellular processes including cell proliferation, migration, and apoptosis [8,9]. The aberrant expression and/or dysfunction of ion channels can impair normal cellular processes, thus leading to the malignancy of tumor cells [10]. The mammalian TRP (Transient Receptor Potential)—channel superfamily includes ion channels that are widely distributed in various tissues and across several cell types [11,12]. They participate in sensing both endogenous and exogenous stimuli [13,14]. TRPV4 channel is a member of the TRP ion channel subfamily V [15]. It can be activated by several physicochemical stimuli, such as moderate heat (>24 °C), mechanical stimulus, the low potential of hydrogen, and inflammation [16]. It is speculated that the tumor microenvironment can easily activate TRPV4 [17]. TRPV4 plays an important role in regulating cancer metastasis in breast, endometrial, gastric, and colon cancers [17,18,19,20,21,22]. Our research indicated that melanoma possesses a higher abundance of TRPV4 among multiple cancers and the expression of TRPV4 in skin-derived melanoma cell lines is significantly higher compared to the normal epidermal cells. In addition, melanoma rapidly becomes life-threatening once it has spread. For more than 40 years, few treatment options were available, and clinical trials were unsuccessful during that time [23]. Therefore, it is urgent to investigate the role of TRPV4 in melanoma metastasis.

Metastasis is initiated and maintained by signaling pathways that mediate cytoskeletal dynamics in the tumor cells [24]. The dynamics of migration to the surrounding microenvironment are the result of the cytoskeleton interaction with cell surface receptors [25]. Tumor cells migrate by binding to the extra-cellular matrix due to actin polymerization which provides the pushing force for protrusion of the leading edge to establish the direction of migration [26]. Many actin-binding proteins are involved in each step of the motility cycle. Among them, cofilin has a central role in controlling actin dynamics [27]. It is active at the leading-edge protrusion of migrating cells and is inactivated upon Ser3 phosphorylation [28]. Cortactin is an important upstream molecule that regulates the state of cofilin; it severs cofilin activity, thereby stabilizing the invadopodia in the dephosphorylated state. Phosphorylation of cortactin is required to sever actin polymerization and release cofilin [29]. Recent studies have shown that TRPV4 can regulate Rho family GTPase to change the cytoskeleton structure and inhibit endometrial cancer metastasis [19]. However, the biological effects of TRPV4 on the key proteins cofilin and cortactin have not been reported yet. Additionally, whether melanoma metastasis can be inhibited in vivo and in vitro by blocking the TRPV4 channel remains unknown.

In addition to physicochemical stimuli, TRPs can also be regulated by some small molecule compounds, such as capsaicin which is an agonist of TRPV1, while menthol is the agonist of TRPM8 [30,31]. Baicalin is obtained from *Scutellaria baicalensis Georgi*, a widely used traditional herbal medicine with various pharmacological effects including antitumor functions [32]. It can suppress the migration and invasion of human glioblastoma cells in a Ca^2+^-dependent manner [33]. Previous investigations have validated the anti-tumor property of Baicalin [34]. Our previous study showed that Baicalin has similar chemical structures to TRPV4 inhibitor-HC067047, which could abrogate the calcium influx induced by TRPV4 agonist GSK1016790A [35]. However, the underlying mechanism in conjunction with TRPV4 and melanoma remains unknown.

In this study, we examined the role of TRPV4 in melanoma metastasis. Our results showed that TRPV4-induced cytoskeleton reorganization is critical for melanoma metastasis. Our previous studies have shown that Baicalin, from the natural product library, was a potent TRPV4 antagonist. Recently, we confirmed that Baicalin significantly suppressed A375 cell migration, reduced the liver metastases nodes, and prevented B16F10 melanoma cell metastasis. The Src-cofilin pathway played a vital role in TRPV4-induced melanoma metastasis. Collectively, our findings found that TRPV4 is functionally overexpressed in melanoma and established the principle of targeting TRPV4 to inhibit metastasis, as well as revealed that Baicalin might serve as a natural small molecule inhibitor of TRPV4 that may be further optimized to be a therapeutic agent to attenuate metastasis of melanoma.

## 2. Results

### 2.1. High TRPV4 Expression in Melanoma

**Figure 1:**
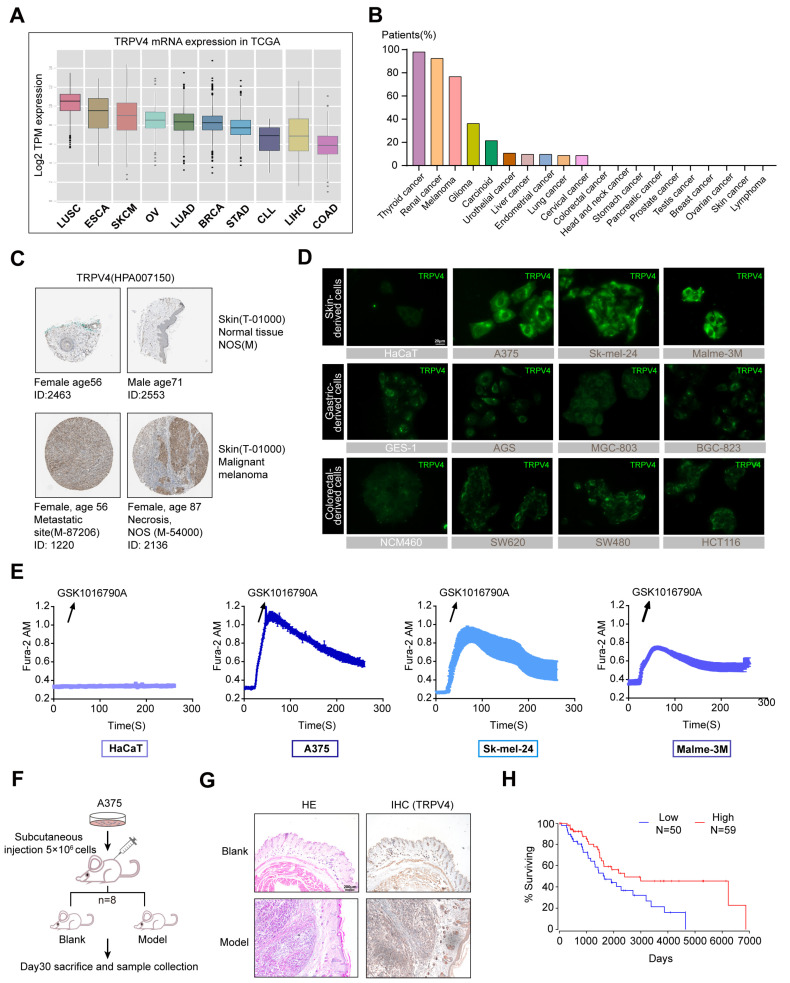
Up-regulation of TRPV4 in melanoma. (**A**) *TRPV4* mRNA expression level in different cancer tissues in TCGA. (**B**) TRPV4 protein expression level in different human cancerous and adjacent non-cancerous/normal tissue in terms of The Human Protein Atlas. (**C**) Strong immunoreactivity was observed in melanoma patients. (**D**) Representative images of TRPV4 expression (green) in multiple cells (Above: Skin-derived cell lines, HaCaT, A375, SK-mel-24, Malme-3M; Middle: Gastric-derived cell lines, GES-1, AGS, MGC-803, BGC-823; Below: Colorectal-derived cell lines, NCM460, SW620, SW480, HCT116). 400×. Scale bar, 20 μm. (**E**) Calcium imaging of HaCaT, A375, SK-mel-24, and Malme-3M cells treated with 2 nM GSK1016790A (n = 6, each group). (**F**) BALB/c-Nude mice were s.c. engrafted with 5 × 10^6^ A375 cells. (**G**) Representative images of HE and IHC (TRPV4) in tumor and normal skin tissues. 40×. Scale bar, 200 μm. (**H**) Survival cox regression data for TRPV4 from SKCM patients using OncoLnc (*p*-value = 0.0176). Data are expressed as means ± SD.

To investigate the expression profile of TRPV4 in different cancers, we analyzed The Cancer Genome Atlas (TCGA) datasets and acquired results indicating that Skin Cutaneous Melanoma (SKCM) possesses a higher abundance of *TRPV4* among multiple cancers (Figure 1A). By further assessing the cytoplasmic immunoreactivity in human cancerous and adjacent non-cancerous/normal tissue in terms of The Human Protein Atlas (https://www.proteinatlas.org/, accessed on 1 October 2021), we were impressed by the situation of melanoma at a positive rate with 9 of 12 patients showing high/medium expression (Figure 1B). Strong staining for TRPV4 was found in pathological tissues of malignant melanoma patients when compared with normal skin tissues (Figure 1C). It has been verified that the protein expression of TRPV4 in skin-derived melanoma cell lines (A375, Sk-mel-24, Malme-3M) was significantly higher when compared to the normal epidermal cells (HaCaT) in our previous study [35]. We further assessed TRPV4 expression in cells by immunofluorescence and the obtained results are consistent with previous immunoblotting (Figure 1D). Our results showed that TRPV4 expression in melanoma cells (HaCaT, A375, Sk-mel-24, Malme-3M) was indeed higher when compared to the normal epidermal cell line (HaCaT), as well as gastric-derived cells (GES-1, AGS, MGC-803, BGC-823) and colorectal-derived cells (NCM460, SW480, SW620, HCT116) (Figure 1D). Intracellular calcium mobilization is widely considered to be the key effect of TRPV4 activation [36]. To determine whether TRPV4 is functionally expressed in melanoma cells, we performed Ca^2+^ imaging to examine the fluctuation of intracellular Ca^2+^ after TRPV4 activation. GSK1016790A, a selective agonist of TRPV4 [37], was used to assess the functional effects of TRPV4 activation. Fura-2 imaging of Ca^2+^ activity showed that GSK1016790A produced rapid and sustained intracellular Ca^2+^ elevation in melanoma cells, and the level of intracellular Ca^2+^ elevation was positively correlated with the expression of TRPV4 (Figure 1E). Subsequently, A375 was selected for subcutaneous in situ tumor modeling for its highest expression of TRPV4, and the intratumoral expression of TRPV4 was obviously higher than in normal skin tissues (Figure 1F,G). To facilitate and advance our understanding of the significance of TRPV4 in melanoma, we extensively explored survival cox regression data for TRPV4 from SKCM patients using OncoLnc (http://www.oncolnc.org/, accessed on 1 October 2021), which was analyzed based on TCGA [38]. It is apparent that patients with lower TRPV4 expression tend to enjoy better prognoses and extended life spans (Figure 1H). Taken together, TRPV4 showed abnormally high functional expression in melanoma cell lines.

### 2.2. Functional TRPV4 Is Required for A375 Metastasis

**Figure 2:**
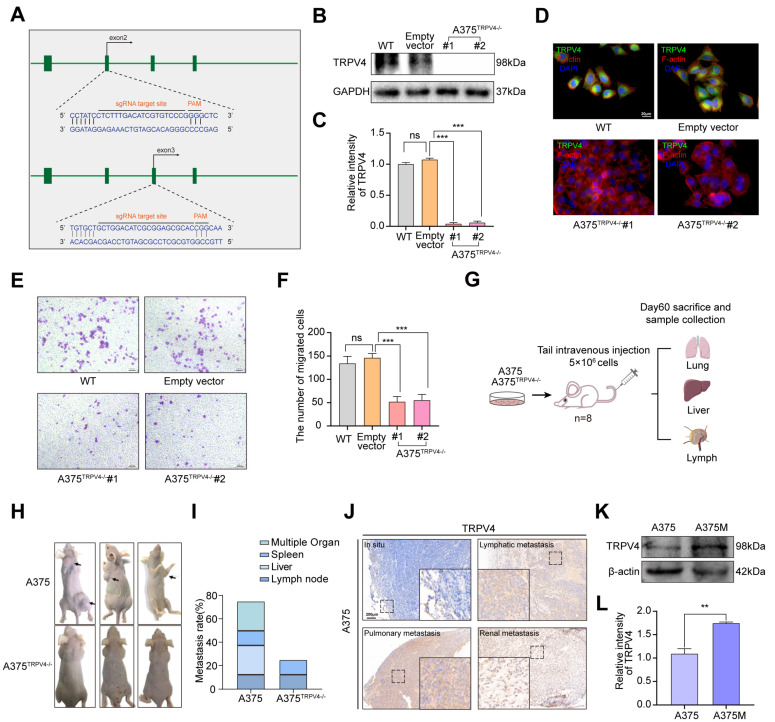
Functional TRPV4 is required for A375 metastasis. (**A**) A375^TRPV4−/−^ cells were produced by the CRISPR/Cas9 system. (**B**,**C**) Representative images of immunoblot analysis of TRPV4 in A375 and A375^TRPV4−/−^ cells. (**D**) Representative images of TRPV4 expression (green) in A375 and A375^TRPV4−/−^ cells. Nuclei were stained with DAPI (blue) and Cytoskeleton was stained with Phalloidin (red). 400×. Scale bar, 20 μm. (**E**,**F**) Measurement of A375 and A375^TRPV4−/−^ cell migration ability using trans-well system. Views were selected randomly from each sample and migrated cells were counted (three views were randomly selected for each group). 100×. Scale bar, 200 μm. (**G**–**I**) Metastasis rate of mouse-injected A375 or A375^TRPV4−/−^ cells were analyzed at the endpoint, the arrow indicates the metastatic tumor in Figure (**H**). (**J**) Representative images of TRPV4 expression in situ, lymphatic metastasis, pulmonary metastasis, and renal metastasis. 40×. Scale bar, 200 μm. (**K**,**L**) Representative images of immunoblot analysis of TRPV4 in A375 and A375M cells. Data are expressed as means ± SD. ** *p* < 0.01, *** *p* < 0.001, ns: not statistically.

Metastasis is the leading cause of death in patients with advanced melanoma [39]. To determine the specific role of TRPV4 in melanoma metastasis induced in A375, Clustered Regularly Interspaced Short Palindromic Repeats (CRISPR)/CRISPR-associated (Cas) protein 9 (CRISPR/Cas9) lentiviral system was used to establish an A375 cell line with knocked out TRPV4, hence referred to as A375^TRPV4−/−^ (Figure 2A). The immunoblotting and immunofluorescence assays validated the complete silencing of the TRPV4 protein (Figure 2B–D). In addition, deletion of the TRPV4 protein significantly interfered with the migration of A375, as indicated by the dramatic reduction in the number of cells on the external surface of the top chamber (Figure 2E,F). Based on our results, we intravenously injected A375 and A375^TRPV4−/−^ into nude mice. Animals were sacrificed after sixty days, and the metastases rates were assessed. Indeed, in vivo TRPV4 deficiency inhibited the invasion and migration of melanoma cells (Figure 2G,H). We speculated that the tumor cells that colonized in the metastatic site had a stronger invasion ability than those in the in situ conditions. Previous in vivo experiments suggested that exposure to A375 via tail injection could cause multi-metastases involving the lymph nodes (Figure 2I). Additionally, compared to in situ conditions, TRPV4 was highly expressed at metastatic sites including kidneys, lymph nodes, and lungs (Figure 2J). Therefore, we isolated and obtained A375 Metastasis (A375M) cells from the lymph nodes of the metastatic model. Our findings indicated that A375M cells possessed relatively enhanced TRPV4 protein expression by western blot analysis (Figure 2K,L). Results of trans-well and wound-healing also proved that A375M cells earn more ability of migration and invasion than A375 cells (Supplementary Materials Figure S1A–D).

### 2.3. A Potent TRPV4 Inhibitor from the Natural Product Library: Baicalin

**Figure 3:**
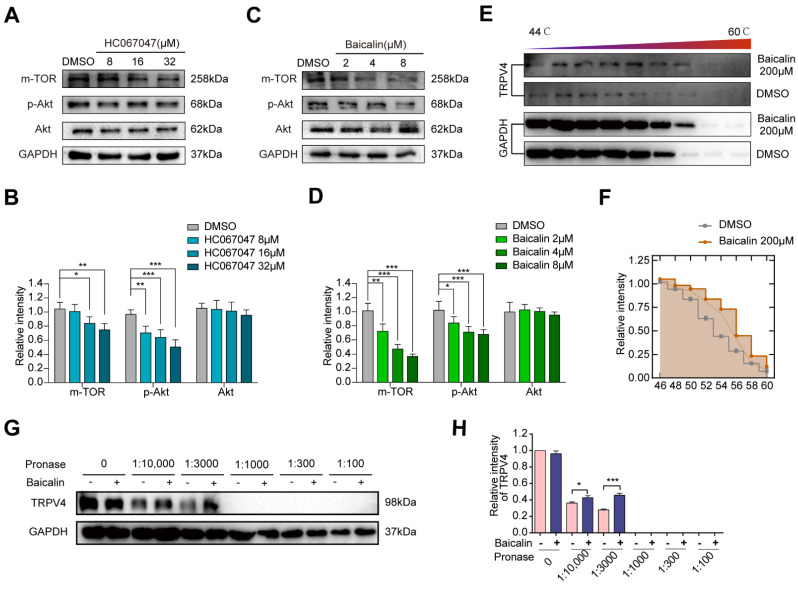
Baicalin is a potent TRPV4 inhibitor. (**A**,**B**) Representative images of immunoblot analysis of mTOR, p-Akt, and Akt in A375 cells treated with HC067047 for 24 h. (**C**,**D**) Representative images of immunoblot analysis of mTOR, p-Akt, and Akt in A375 cells treated with Baicalin for 24 h. (**E**,**F**) CETSA using intact cells, which were exposed to 200 μM Baicalin. (**G**,**H**) Baicalin promotes TRPV4 protein resistance to proteases (DARTS). Data are expressed as means ± SD. * *p* < 0.05, ** *p* < 0.01, *** *p* < 0.001.

Since TRPV4 regulated the cell invasion and migration in melanoma, we tried to inhibit the malignancy by expression reduction and activity inhibition of TRPV4. Natural products are important in the development of anticancer drugs; they possess several characteristics including structural diversity, lower toxicity, and fewer side effects [40]. We performed the DISCOVERY STUDIO 4.0 (Discovery Studio™: Île-de-France, France) and found that Baicalin was structurally similar to HC067047 (selective TRPV4 antagonist) and could potentially directly bind to TRPV4. As the activation of TRPV4 is mainly characterized by calcium influx, Ca^2+^ imaging was used to assess the significance of Baicalin-mediated blocking of TRPV4 activation. Our existing results showed that Ca^2+^ influx induced by GSK1016790A could be abolished with 8 μM Baicalin treatment [35]. We further evaluated the effect of Baicalin on the expression of TRPV4 in A375 and found that Baicalin did not affect the protein expression of TRPV4. However, the expression of the mammalian Target of Rapamycin (mTOR) and phosphor-serine/threonine kinase Akt (p-Akt) downstream of Ca^2+^ influx was markedly decreased after Baicalin treatment, consistent with observations in the HC067047 treatment group by western blot analysis (Figure 3A–D). Since TRPV4 is a Ca^2+^ permeable channel, we examined if the above functions of Baicalin were associated with its ability to bind to and directly inhibit TRPV4 channel activation. To verify the direct binding ability of Baicalin to TRPV4, we used two recently developed, label-free, biophysical assays: CETSA and DARTS [41,42]. As shown in Figure 3E,F, Baicalin could increase the heat stability of TRPV4 protein compared with the control group. Similarly, Baicalin could also increase the enzymolysis resistance of TRPV4 protein compared with the control group (Figure 3G,H), all of which confirmed the potential direct Baicalin binding to TRPV4. Thus, our results showed that Ca^2+^-permeable TRPV4 is functionally and aberrantly expressed in melanoma cell lines, and its activation could be inhibited by Baicalin treatment.

### 2.4. In Vitro and In Vivo Effects of Baicalin on Melanoma Metastasis

**Figure 4:**
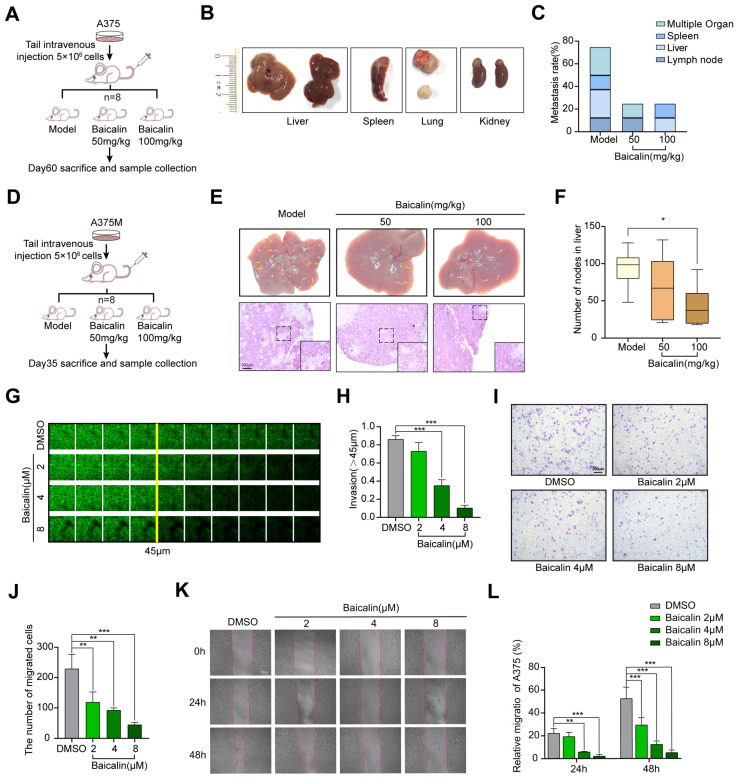
Baicalin inhibits A375 metastasis. (**A**) BALB/c-Nude mice were i.v. engrafted with 5 × 10^6^ A375 cells (n = 8, each group). (**B**) Metastatic colonies formed in the liver, spleen, kidney, and lymph node 60 days after A375 melanoma cells injection, with 50 mg/kg, 100 mg/kg Baicalin or saline. (**C**) Metastasis rate of mouse injected A375 cells treated with or without Baicalin. (**D**) BALB/c-Nude mice were i.v. engrafted with 5 × 10^6^ A375M cells (n = 8, each group). (**E**) Metastatic colonies formed in the liver at 21 days and mice were sacrificed at day 35 after A375M melanoma cell injection, with 50 mg/kg, 100 mg/kg Baicalin or saline treatment. HE staining patterns showed blue. 40×. Scale bar, 200 μm. (**F**) The number of metastatic nodes was counted. (**G**,**H**) Measurement of A375 cell invasion ability using 3D stroma invasion assay with or without incubating with Baicalin for 24 h. (**I**,**J**) Measurement of A375 cell migration ability using the trans-well system with or without incubating with Baicalin for 24 h. Views were selected randomly from each sample and migrated cells were counted (three views were randomly selected for each group). 100×. Scale bar, 200 μm. (**K**,**L**) Measurement of A375 and A375 cell migration ability using a wound-healing assay with or without incubating with Baicalin for 24 h. Views were selected randomly from each sample. 100×. Scale bar, 200 μm. Data are expressed as means ± SD. * *p* < 0.05, ** *p* < 0.01, *** *p* < 0.001.

To further validate the role of Baicalin in melanoma metastasis, we performed in vivo metastatic experiments. We observed that the Baicalin treatment suppressed tumor metastasis when compared to the control group. The metastatic nodules on the lung, liver, spleen, and lymph nodes were counted 60 days after the injection, and we found that the mice treated with 100 mg/kg Baicalin had a lower metastasis rate (Figure 4A–C). A375M cells were injected intravenously into nude mice and three weeks after injection, the metastatic nodules on the liver sections of tumor xenografts were observed. We found that the mice treated with Baicalin had fewer liver metastases compared to the control group; histological photomicrographs of H&E stained liver tissue sections further confirmed our results (Figure 4D–F). To exclude the possibility of the specific effect of Baicalin in human melanoma cell lines, we performed the in vivo metastasis experiment in C57BL6 using B16F10, which also expresses TRPV4. Consistent with our previous results, mice treated with Baicalin had significantly lower lung metastases (Supplementary Materials Figure S2A–C). Collectively, these findings suggested an important role of Baicalin in the in vivo inhibition of melanoma metastasis. Next, we evaluated the effect of Baicalin on migration and invasion of A375 cells by 3D stroma invasion, trans-well, and wound-healing assays. As shown in Figure 4G,H, Baicalin treatment significantly reduced the invasion ability of A375 cells. It also decreased the migration ability of the cells (Figure 4I–L). Similar results were obtained in different melanoma cells treated with Baicalin (8 μM) by trans-well) (Supplementary Materials Figure S3A). Homoplastically, HC067047 treatment also significantly reduced the migration and invasion ability of A375 cells (Supplementary Materials Figure S3B–E).

### 2.5. TRPV4 Promotes Melanoma Metastasis by Regulating Cell Motility

**Figure 5:**
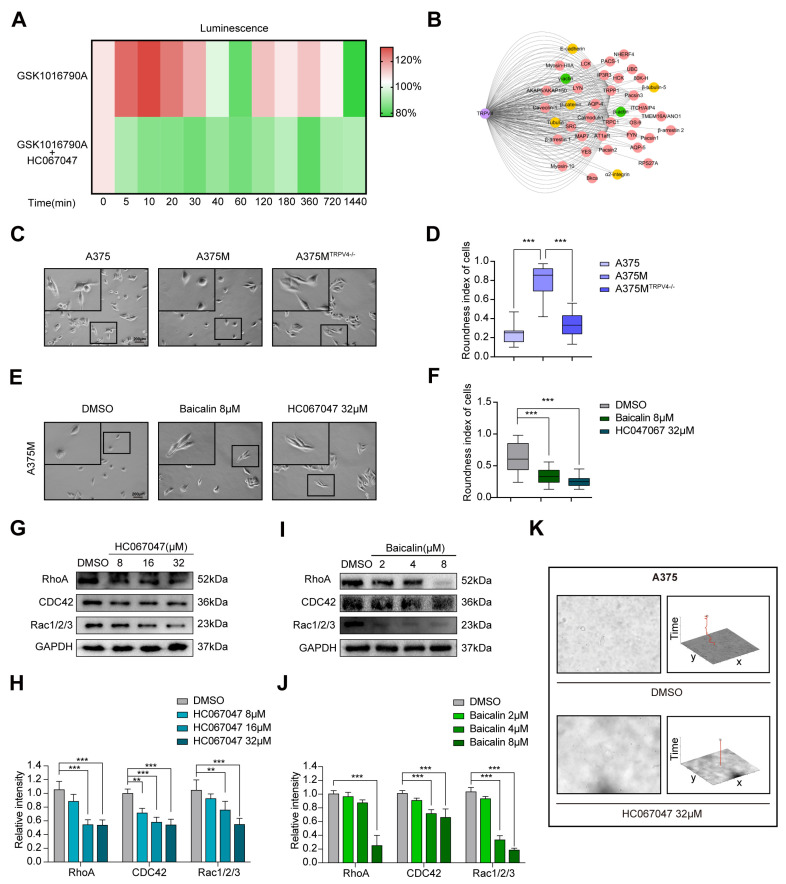
TRPV4 induces cell motility change through cytoskeletal rearrangement. (**A**) Luminescence intensive detection of ATP in A375 cells treated with GSK1016790A (above) and HC067047 (below). (**B**) Protein interaction network of TRPV4. (**C**) Representative bright-field images of A375, A375M, and A375^TRPV4−/−^ cells. 200×. Scale bar, 200 μm. (**D**) Cell morphology (roundness index) of A375, A375M, and A375^TRPV4−/−^ cells. (**E**) Representative bright-field images of A375M cells treated with Baicalin or HC067047. 200×. Scale bar, 200 μm. (**F**) Cell morphology (roundness index) of A375M, and A375M cells treated with Baicalin or HC067047. (**G**,**H**) Representative images of immunoblot analysis of RhoA, CDC42, and Rac1/2/3 in A375 cells treated with HC067047 for 24 h. (**I**,**J**) Representative images of immunoblot analysis of RhoA, CDC42, and Rac1/2/3 in A375 cells treated with Baicalin for 24 h. (**K**) Cell trajectories on phase contrast images (cell trajectories are marked in red). Data are expressed as means ± SD. ** *p* < 0.01, *** *p* < 0.001.

Further investigation of the mechanism behind TRPV4 in regulating melanoma metastasis was arranged. We first performed apoptosis-related protein detection, cell cycle analysis using Propidium Iodide (PI) staining, and Annexin-V/PI apoptosis detection by flow cytometry for the A375 cells. These results all showed that treatment with HC067047 or Baicalin could not induce apoptosis or change cell cycle status in A375 cells (Supplementary Materials Figure S4A–E). Changes in intracellular Ca^2+^ influx led to the alteration in the levels of cytosolic Adenosine Triphosphate (ATP), which in turn regulates cytoskeletal dynamics and cellular morphology [43,44]. Cellular ATP levels in A375 were measured using a bioluminescent assay kit and increased continuously with TRPV4 agonist GSK1016790A, which was inhibited by HC067047 (Figure 5A). TRIP database analysis indicated that TRPV4 interacted extensively with proteins related to the cytoskeleton and cell motility, especially Src family proteins which are the other group of nonreceptor tyrosine kinases involved in the regulation of various signaling pathways that promotes cell motility (Figure 5B). To leave the primary tumor site, cancer cells disseminate using different migration modes, such as rounded-amoeboid or elongated-mesenchymal routes, which allow their transport both locally and to distant sites along with invasion through basement membranes. The highly metastatic melanoma cells have a more rounded morphology [45]. We found that A375M cells have a more rounded morphology compared to the A375 and A375M^TRPV4−/−^ cells (Figure 5C,D). To investigate the effect of TRPV4 on cytoskeleton reorganization, A375M cells were treated with HC067047 or Baicalin. As shown in Figure 5E,F, A375M treated with HC067047 or Baicalin was less rounded when compared to the control. As the Rho family of small GTPases, including CDC42, Rac1/2/3, and RhoA, are key regulators of both cell motility and the cytoskeleton, we assessed RhoA, CDC42, and Rac1/2/3 expression levels using western blotting (Figure 5G–J). It is obvious that both HC067047 and Baicalin could decrease the protein expression of Cdc42, Rac1/2/3, and RhoA, which further certifies a close relationship between the TRPV4 and cell motility. We also measured the locomotion of A375 treated with HC067047 and found that HC067047 significantly inhibited the motility of A375 (Figure 5K). These results indicated that TRPV4 enhanced A375 cell metastasis by inducing changes in the cell morphology and motility, but not cell apoptosis.

### 2.6. TRPV4 Regulates Melanoma Metastasis by Inducing Morphological Changes in Cells through the Src-Cofilin Axis

**Figure 6:**
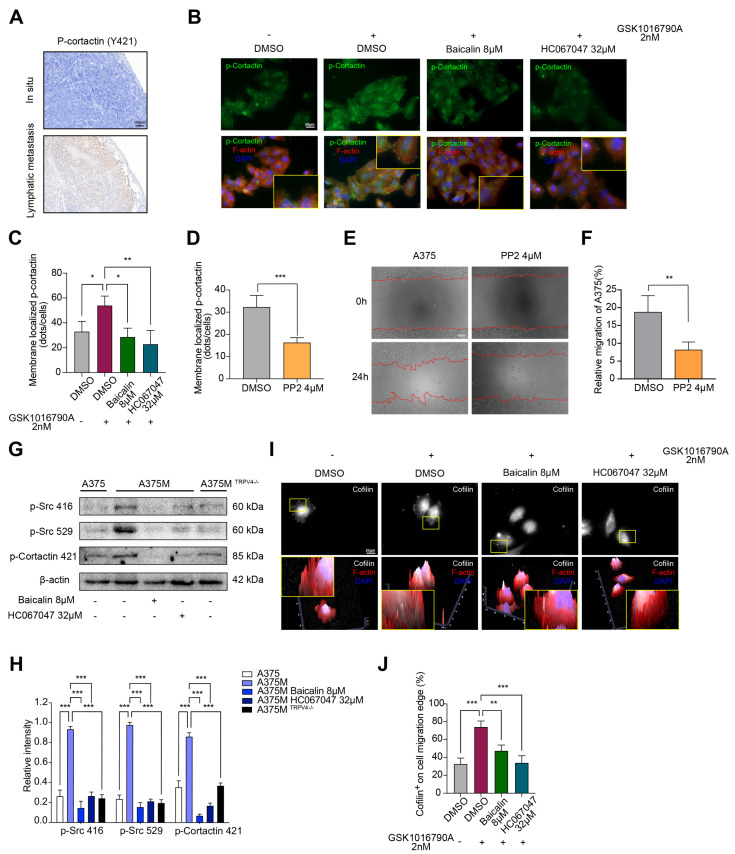
TRPV4 regulates the Src-cofilin axis. (**A**) Representative immunohistochemistry images of TRPV4. 40×. Scale bar, 200 μm. (**B**) Representative images of p-cortactin expression (green) in A375 cells treated with GSK1016790A, HC067047, or Baicalin for 24 h. Nuclei were stained with DAPI (blue), Cytoskeleton were stained with Phalloidin (red). 400×. Scale bar, 20 μm. (**C**) Membrane localized p-cortactin in A375 cells treated with GSK1016790A, HC067047, or Baicalin for 24 h. (**D**) Membrane localized p-cortactin in A375 cells treated with PP2 for 24 h. (**E**,**F**) Representative images of A375 cells migration at 24 h after treated with PP2. 100×. Scale bar, 200 μm. (**G**,**H**) Representative images of immunoblot analysis of p-Src416, p-Src529, p-Cortactin421 in A375, A375M, and A375M^TRPV4−/−^ cells treated with Baicalin or HC067047 for 24 h. (**I**,**J**) Representative images and analysis of cofilin expression on cell migration edge (white) in A375 cells treated with GSK1016790A, HC067047, or Baicalin. Nuclei were stained with DAPI (blue), Cytoskeleton were stained with Phalloidin (red). 400×. Scale bar, 20 μm. Data are expressed as means ± SD. * *p* < 0.05, ** *p* < 0.01, *** *p* < 0.001.

Activation of Src family kinases, mainly induced by Tyr416 residue phosphorylation, directly or indirectly promotes the phosphorylation and activation of downstream targets, and Cortactin is the most relevant downstream protein for cytoskeleton remodeling and migration [46]. Consequently, to further elucidate the underlying mechanism of TRPV4-mediated cell morphological changes, we evaluated the effects of TRPV4 on the activation of cortactin and we found that the cancer metastatic sites formed by A375 cells show higher levels of cortactin phosphorylation levels compared with in situ cancer tissue (Figure 6A). The immunofluorescence results showed that the activation of TRPV4 in A375 increased the phosphorylation of cortactin, while this effect can be reversed by HC067047 and Baicalin (Figure 6B,C). Consistent with this, the immunofluorescence assay found that the depletion of TRPV4 in A375 cells decreased the phosphorylation of cortactin (Supplementary Materials Figure S5A). To find out whether Src kinase plays a major role in it, A375 cells treated with PP2 (an inhibitor of Src kinases) were used for further study. We found that PP2 could significantly decrease the phosphorylation of cortactin (Figure 6D) and inhibit the migration in A375 cells (Figure 6E,F). Based on these findings, we hypothesized that TRPV4 could likely regulate tumor metastasis through Src-cortactin signaling. Therefore, we assessed p-Src416, p-Src529, and p-Cortactin expression levels by western blot analysis. The results showed that A375M cells had higher p-Src416, p-Src529, and p-Cortactin levels, and HC067047 and Baicalin could significantly decrease these levels in A375M cells (Figure 6G,H). In A375M^TRPV4−/−^ cells, the expression of p-Src416, p-Src529, and p-Cortactin is obviously lower compared to A375M cells (Figure 6G,H). Since the phosphorylation of cortactin is required to release cofilin to sever actin polymerization, we also examined the effect of HC067047 or Baicalin on cofilin aggregation. The results of immunofluorescence assays showed that the HC067047 or Baicalin treatment could inhibit the aggregation of cofilin on the cell migration edge (Figure 6I,J); HC067047 promoted the phosphorylation of cofilin (Supplementary Materials Figure S5B). Overall, these results suggested that TRPV4 regulated melanoma metastasis by inducing cell morphological changes through the Src-cofilin axis. Baicalin was a potent inhibitor of TRPV4.

## 3. Discussion

In this study, we investigated the effect of TRPV4 on melanoma metastasis. The animal experimental model we constructed using two cell lines jointly validated that HC067047 treatment significantly decreased metastases of melanoma cells. We also obtained A375M cells from lymph metastases nodes and generated an A375 TRPV4 knock-out (KO) cell line to validate the pro-metastasis effect of TRPV4. We hypothesized that Baicalin could inhibit melanoma metastasis in a TRPV4-dependent manner. We tried to better understand the underlying mechanism and found that Baicalin inhibited the calcium influx induced by TRPV4 activation and induced the cell morphological changes by cytoskeletal rearrangements. Furthermore, Baicalin decreased the levels of p-Src416, p-Src529, and p-Cortactin in A375M cells and inhibited the aggregation of cofilin on the cell migration edge. These results suggest that Baicalin inhibited melanoma metastasis by regulating the TRPV4-Src-cofilin axis.

Metastasis is a complicated multistep process involving an “invasion-metastasis cascade” and is the leading cause of mortality in cancer patients [47,48]. Tumor cells require altered cell morphologies and modes of migration to successfully establish metastases sites [49,50,51]. In this study, we specifically focused on the rounded-amoeboid or elongated-mesenchymal migratory phenotypes for local invasion in the tumor-surrounding tissue. We showed that A375M derived from metastases sites are highly malignant and are more rounded than A375. Baicalin reduced the roundness index in A375M cells. Rho family proteins including RhoA, CDC42, and Rac are key regulators of the actin cytoskeleton and play an important role in tumor metastasis [52,53]. In this study, we investigated the effect of Baicalin on the expression of RhoA, CDC42, and Rac1/2/3 by western blot analysis. The results show that Baicalin reduced their expression, which suggests the actin cytoskeleton regulation role of Baicalin.

Recently, it was reported that tumor cells maintain single-cell polarity in the liquid phase, unlike the amoeboid migration, and thus determine the metastasis outcome [54]. Tumor cells in the bloodstream exert cortical ezrin-rich poles to adhere to vascular endothelium and subsequently facilitate the metastases processes. This study used an orthotopic tumor model by a subcutaneous injection of A375 melanoma cells. However, we found liver metastasis in only one of eight mice at eight weeks. Thus, we decided to use the hematogenous metastasis mouse model for further experiments in this study.

The current study has a few limitations. We only used a hematogenous metastasis mouse model in this study. When tumor cells are intravenously injected into the mice, circulating melanoma cells need to attach to the endothelium, and subsequently, trans-endothelial migration occurs. These are critical steps in metastasis. Baicalin may also regulate the single-cell polarity of melanoma cells in the bloodstream or exert its effect on the endothelium to influence the later steps of metastasis. Given the essential roles of Rho family GTPases in actin cytoskeleton organization, it is likely they are also involved in establishing or stabilizing single-cell polarity. Future studies are thus needed to better understand the effects of Baicalin on melanoma metastasis in blood circulation. Additionally, we have not measured the pharmacokinetics and pharmacodynamics of Baicalin to evaluate the dosage sufficiency and safety of Baicalin, and these need to be tested. In addition, whether TRPV4 induces Src activation via ATP has not been carefully verified.

In summary, our findings may have implications for the potential role of TRPV4 in melanoma metastasis. Baicalin could decrease melanoma metastasis by regulating cell motility and the mechanism of anti-tumor metastasis is related to blocking TRPV4 activation.

## 4. Materials and Methods

### 4.1. Cell Lines and Cell Culture

The melanoma cell lines B16F10, A375, SK-mel-24, Malme-3m, gastric cancer cell lines AGS, MGC-803, BGC-823, colorectal cancer cell lines SW480, SW620, HCT116, human gastric mucosa cell line GES-1, human colonic epithelial cell line NCM460, and human keratinocyte cell line HaCaT were obtained from laboratory stocks in Nanjing University of Chinese Medicine and were cultured in DMEM (Gibco, MA, USA), MEM (Gibco, MA, USA), EMEM (Gibco, MA, USA), or RPMI 1640 (Gibco, MA, USA) medium, supplemented with 10% Fetal Bovine Serum (FBS), penicillin (Amresco, Houston, TX, USA) (100 U/mL), and streptomycin (100 μg/mL). A375M was derived from lymphoma metastasis tissue in a mouse model intravenously injected with A375 cancer cells. They were incubated at 37 °C and 5% CO_2_. TRPV4-KO-A375 cells were generated by CRISPR/Cas9 gene-editing technology using commercial plasmid (PX459, Addgene, Boston, MA, USA). Each CRISPR/Cas9 KO product consisted of a mixture of plasmid and guide RNA designed for improved KO efficiency. gRNA sequences were derived from the CRISPR Cas9 protein, which was directed to induce a site-specific double-strand break in the genomic DNA. A375 cells were transfected using an X-tremeGENETM HP DNA reagent (Roche Diagnostics, Indianapolis, USA). The CRISPR cells were polyclonal. Single-cell TRPV4 KO clones were isolated from gRNA-transduced populations. Two clones of A375 were expanded, selected, and identified by immunoblotting. The verified cell clone having the gene KO was used for further experiments.

### 4.2. Animals

Six-week-old male C57BL/6 mice and BALB/c nude mice were purchased from Charles River (Beijing, China). Animals were maintained in a controlled environment at a temperature of 23 ± 1 °C with a 12–12 h light-dark cycle (light cycle, 07:00–19:00) and fed a standard diet. Water was freely available. All experimental protocols using animals were approved by the Committee on Laboratory Animal Care of the Nanjing University of Chinese Medicine, the ethical approval code numbers are ACU171111 and ACU170502. All the animals were given humane care according to the guidelines of the National Institutes of Health (USA).

### 4.3. Experimental Metastasis Model and Treatment Studies

To create an experimental model of metastasis, A375, A375^TRPV4−/−^, and A375M human melanoma cells at a density of 5 × 10^6^ cells/animal were intravenously injected into BALB/c nude mice. In the treatment regime, 50 mg/kg, 100 mg/kg Baicalin (MCE, Monmouth Junction, UT, USA) or saline was administered through intraperitoneal injection. At the end of the experiment, the mice were euthanized, the images of organs were captured, and the liver metastases nodules were counted by a stereoscopic microscope (Zeiss, Germany). Additionally, B16/F10 mouse melanoma cells were intravenously injected at a density of 1 × 10^6^ cells/animal into C57BL/6 mice. In the treatment regime, 30 mg/kg, 60 mg/kg Baicalin or saline was administered through intraperitoneal injection. Twenty days after injection, the mice were euthanized, and the nodules (corresponding to pulmonary metastases of melanoma cells) were counted under a stereoscopic microscope.

### 4.4. Ca^2+^ Imaging

A total of 5 × 10^4^ cells were seeded on coverslips and incubated with Fura-2 AM (Beyotime, China) at 37 °C for 30 min in the dark. Cells were washed thrice with normal physiological saline solution (NPSS, 140 mmol/L NaCl, 5 mmol/L KCl, 1 mmol/L CaCl_2_, 1 mmol/L MgCl_2_, 10 mmol/L glucose, and 5 mmol/L HEPES, pH 7.4). Fura-2 fluorescence was measured using an Olympus fluorescence imaging system at dual excitation wavelengths of 340 and 380 nm. Changes in the Fura-2 ratio were calculated as the changes in the [Ca^2+^].

### 4.5. Cellular Thermal Shift Assay (CETSA)

A solution of the Baicalin (200 μM) in Phosphate-Buffered Saline (PBS)/dimethyl sulfoxide (DMSO) or PBS/DMSO as the vehicle, was added to the cell extract at a final concentration of 1% PBS/DMSO. Then, the cells of the two groups were incubated for 40 min at 37 °C. After incubation, the two groups were divided into four parts of 100 μL each in 0.2 mL PCR tubes. Thus, one sample contained the compound and the other contained the vehicle. Pairs of one control and one experimental aliquot were heated at 44 °C, 46 °C, 48 °C, 50 °C, 52 °C, 54 °C, 56 °C, 58 °C, 60 °C, or 62 °C for 3 min, followed by a 3 min incubation at room temperature. Subsequently, the extract was centrifuged at 12,000× *g* for 20 min at 4 °C. The supernatant was collected; the protein concentration was determined by the bicinchoninic acid (BCA) (Meridian, TX, USA) method and the proteins were separated by SDS gel electrophoresis for western blot analysis.

### 4.6. Drug Affinity Responsive Target Stability Assay (DARTS)

Cells were collected and lysed in the lysis buffer containing protease and phosphatase inhibitors. Protein concentrations were determined to ensure an equal amount of protein lysate per sample using the bicinchoninic acid protein assay kit (Thermo Fisher Scientific, Meridian, TX, USA). Cell lysates were incubated with Baicalin for 15–30 min using a thermomixer at room temperature. Each cellular lysate sample was proteolyzed at 4 °C for 30 min with different pronase concentrations (1:100, 1:300, 1:1000, 1:3000, 1:10,000) (Roche Diagnostics, Indianapolis, USA). Subsequently, the reaction was suspended in EDTA and incubated on ice for 10 min. The samples were then loaded on SDS-PAGE and the gel was stained with Coomassie Brilliant Blue. Ultimately, the specific gel bands that were seen in treatment samples were compared with controls, cut out, and further processed for western blotting analysis.

### 4.7. Immunofluorescence

Cells were seeded in 12-well plates and cultured in a medium supplemented with 10% FBS for 24 h. After fixing with 4% paraformaldehyde for 30 min, the cells were blocked with 1.5% bovine serum albumin (BSA) for 1 h. Then, cells were incubated with primary antibodies against TRPV4 (1:2000, Abcam, Cambridge, MA, USA), phospo-cortactin (1:1000, Affinity, China), and cofilin (1:1000, Affinity, China) at 4 °C overnight followed by incubation with FITC-labeled goat anti-rabbit IgG for 1 h at room temperature. DAPI (Beyotime, China) was used to stain the cell nucleus. All the images were captured with the fluorescence microscope (Zeiss, Axio vert A1, Germany).

### 4.8. Wound-Healing Assay

Cell migration was evaluated by wounding healing assays. Briefly, A375 cells were plated in 6-well plates (4 × 10^5^ cells/plate). After the cells reached complete confluence, they were starved overnight and scratched with a sterile 200 μL pipette tip. They were washed using PBS buffer, subsequently treated with reagents, and cultured for 24 h. They were imaged before and after 24 h of culture.

### 4.9. Trans-Well Assay

Cell invasion assays were performed in 12-well trans-well units. After incubation at 37 °C for 24 h, the cells were fixed with ethanol and stained with 0.1% crystal violet. The cells that had invaded the lower surface of the membrane were photographed by a Zeiss Microscope at 10× magnification. Random fields were selected for each sample and cells were counted.

### 4.10. Immunohistochemical Staining and Histopathological Analysis

The tissue sections and slides were deparaffinized and hydrated. The antigens were heat-retrieved, followed by tissue blocking; overnight primary antibody incubation at 4 °C; secondary antibody incubation; and incubation with ABC reagent. The slides were scanned using the Fluorescence Inversion Microscope System (Olympus, Japan). The tissue sections (5 mm) were stained with hematoxylin and eosin (H&E). The slides were imaged using the Fluorescence Inversion Microscope System (Olympus, Japan).

### 4.11. Inverted Invasion Assay

In brief, 4 × 10^5^ A375 cells/dish were added with 5 μL dye and incubated at 37 °C for 15 min. After staining, the cells were centrifuged, cleaned twice with PBS, and re-suspended in DMEM complete medium and inoculated in special dishes. After 4 h, the cells adhered to the wall and the supernatant was aspirated. Basal medium: serum: matrix glue = 2:2:1 on 300 μL clear liquid. Baicalin with the final concentration of 8 μM, 16 μM, 32 μM, and DMSO solution was added into the culture supernatant. After culturing for 24 h, confocal microscopes were used to observe and photograph.

### 4.12. SDS-PAGE and Western Blot Analysis

A375 and A375M cells were lysed with RIPA buffer (Beyotime, China), and total proteins of approximately 25 ng were separated by SDS-PAGE and then transferred to polyvinylidene fluoride membranes (Millipore, Billerica, MA, USA). After labeling with antibody and Goat Anti-Rabbit IgG (H þ L) HRP antibodies, the membranes were scanned using a BIORAD imaging system (chemiDOCTMXRS, Bio-Rad, Hercules, CA, USA). Anti-TRPV4 antibody, Akt (pan) (11E7) (CST, Danvers, MA, USA), Rabbit mAb (BOSTER, Chicago, IN, USA), Phospho-Akt (Ser473) (D9E) XP^®^ (CST, Danvers, MA, USA), Rabbit mAb (BOSTER, Chicago, IN, USA), Phospho-Cortactin (Tyr 421), Anti-Bax antibody (CST, Danvers, MA, USA), Anti-Bcl-2 antibody (CST, Boston, MA, USA), Anti-Caspase-3 antibody (CST, Danvers, MA, USA), Anti-Cdc42 antibody (CST, Danvers, MA, USA), Anti-RhoA antibody (CST, Danvers, MA, USA), Anti-Rac1/2/3 antibody (CST, Danvers, MA, USA), Anti-m-TOR antibody (CST, Danvers, MA, USA).

### 4.13. Statistical Analysis

All statistical analyses were carried out with the GraphPad Prism software version (GraphPad, San Diego, CA, USA). Values were expressed as the mean ± SD of at least three independent experiments. One-way analysis of variance (ANOVA) or t-tests were used to compare in groups. The results were considered statistically significant at * *p* < 0.05, ** *p* < 0.01, *** *p* < 0.001.

## 5. Conclusions

In the present study, we found that Baicalin inhibited calcium influx induced by TRPV4 activating and inhibiting melanoma cell migration and invasion. Additionally, we found that Baicalin inhibits melanoma metastasis by modulating TRPV4-Src-cofilin axis signaling. Collectively, targeting TRPV4 is now a promising area of cancer therapy, and Baicalin is a potential therapeutic drug targeting TRPV4 for the prevention and therapy of melanoma metastasis.

## Data Availability

The data supporting the findings of this study are available from the corresponding author upon reasonable request.

## Supplementary Materials

The supporting information can be downloaded at: https://www.mdpi.com/article/10.3390/ijms232315155/s1.
