# Supplementary material for: TRPV4 Promotes Metastasis in Melanoma by Regulating Cell Motility through Cytoskeletal Rearrangement
Source: Int J Mol Sci. 2022 Dec 2;23(23):15155. doi: 10.3390/ijms232315155 (PMC9737014; doi:10.3390/ijms232315155)
Supplement: Supplementary file 1 [file ijms-23-15155-s001.zip › ijms-2056497-supplementary.pdf]

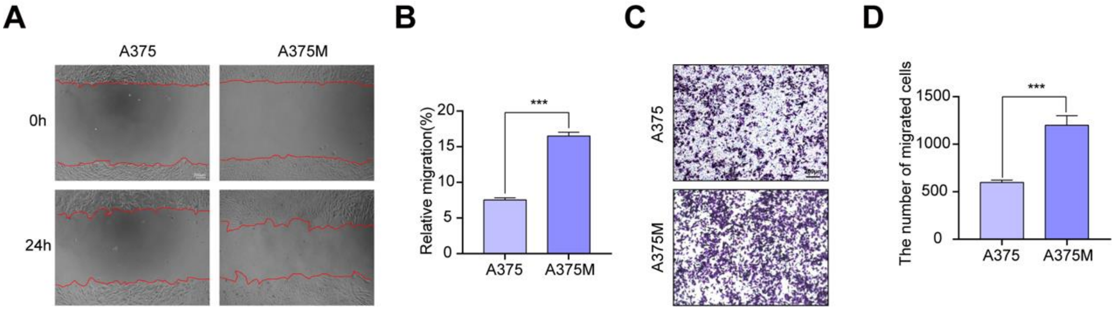

Supplementary Materials Figure S1.

The migration ability of A375 and A375M cells. (A) and (B) The migration ability of A375 and A375M cells was compared by wound-healing assay. 100×. Scale bar, 200 μm. (C) and (D) The migration ability of A375 and A375M cells was compared by a trans-well system. 100×. Scale bar, 200 μm. Data are expressed as means ± SD. ***P < 0.001.

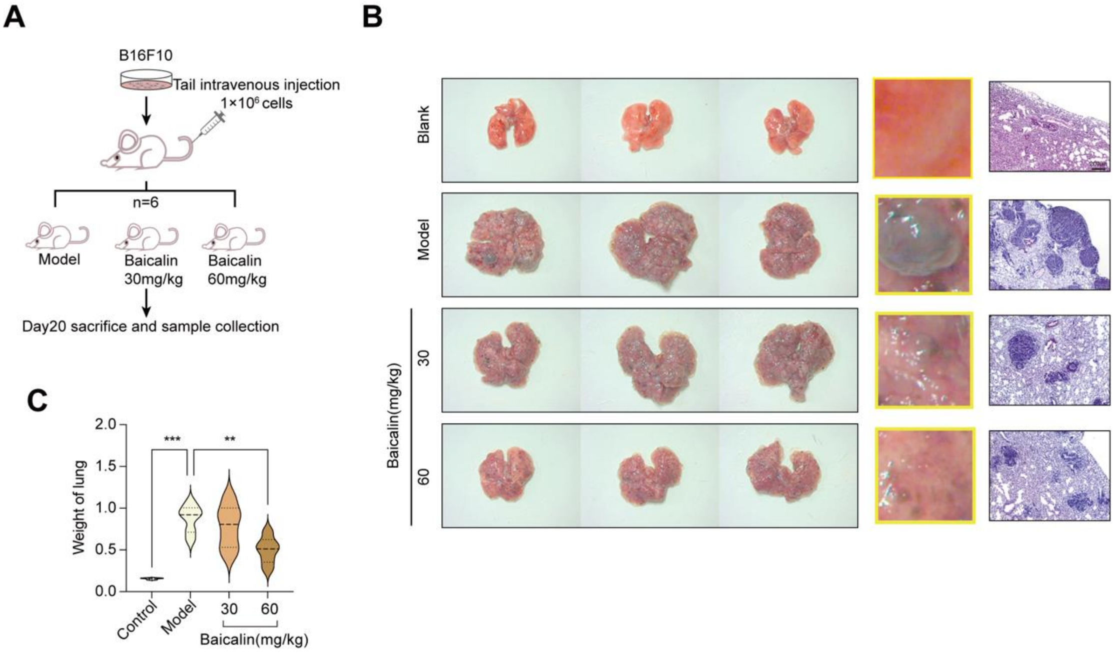

Supplementary Materials Figure S2.

In vivo effects of Baicalin on melanoma metastasis constructed by B16F10 cells. (A) BALB/c-Nude mice were i.v. engrafted with 1×10^6 B16F10 cells. (B) Pulmonary metastasis of mouse injected B16F10 cells treated with 50mg/kg, 100mg/kg Baicalin or without treatment. Hematoxylin and eosin (H&E) staining patterns showed right. 40×. Scale bar, 200 μm. (C) Lung weight statistics of different groups. (n=6, each group). Data are expressed as means ± SD. **P < 0.01, ***P < 0.001.

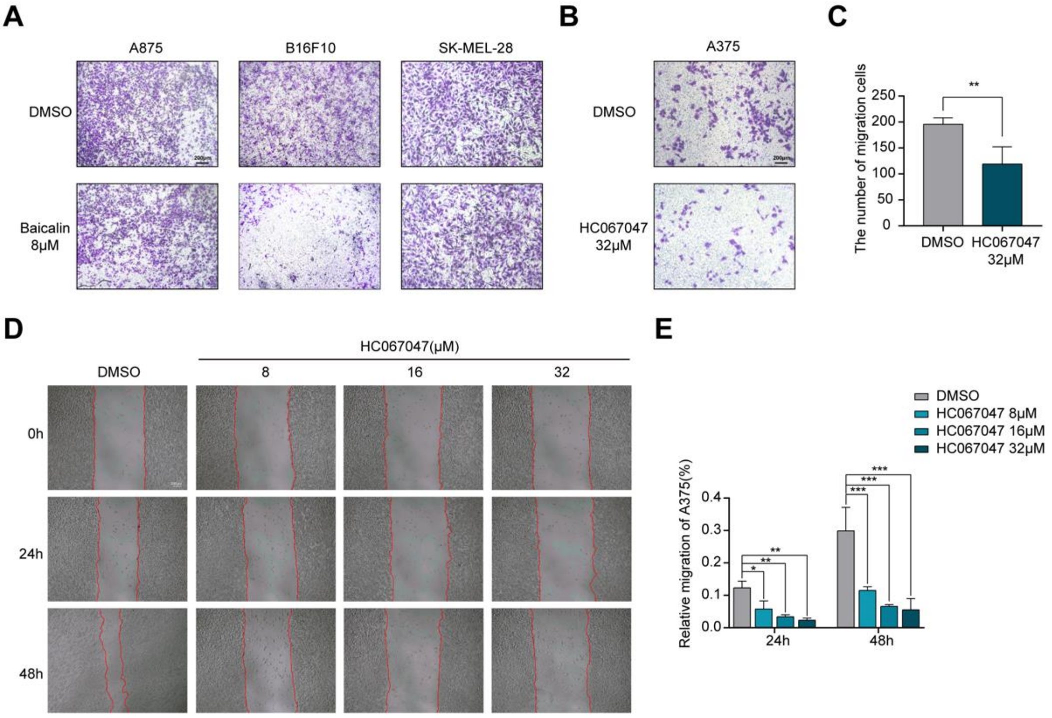

Supplementary Materials Figure S3.

Baicalin inhibit metastasis of different melanoma cells and HC067047 inhibit A375 metastasis. (A) Measurement of A875, B16F10, SK-NEL-28 cells migration ability using trans-well system with or without incubating with Baicalin for 24h. 100×. Scale bar, 200 μm. (B) and (C) Measurement of A375 cells migration ability using a trans-well system with or without HC-067047 for 24h. 100×. Scale bar, 200 μm. (D) and (E) The migration ability of A375 cells was compared by wound-healing assay with or without incubating HC-067047 for 24h. 100×. Scale bar, 200 μm. Data are expressed as means ± SD. *P < 0.05, **P < 0.01, ***P < 0.001.

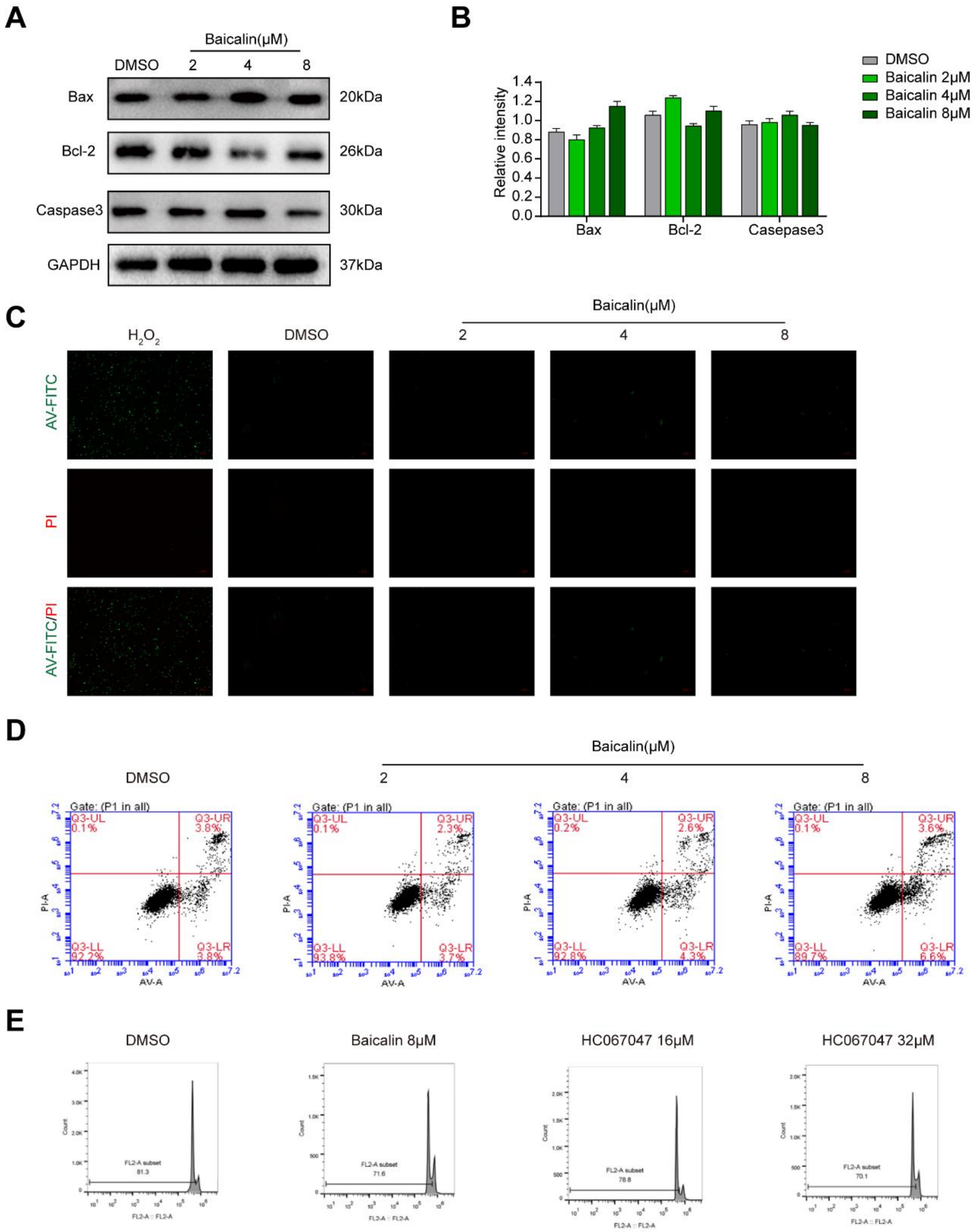

Supplementary Materials Figure S4.

The effect of Baicalin on A375 apoptosis. (A) and (B) Representative images of immunoblot analysis of Bax, Bcl-2 and Caspase3 in A375 treated with Baicalin. (C) Representative images of AV (green) and PI (red) in A375 cells treated with Baicalin. 100×. Scale bar, 50 μm. (D) Flowcytometry dot plots showing % AV+PI+ (Right) A375 cells after treatment with Baicalin. (E) Representative Flowcytometry results of % PI+ A375 cells after treatment with Baicalin. Data are expressed as means ± SD.

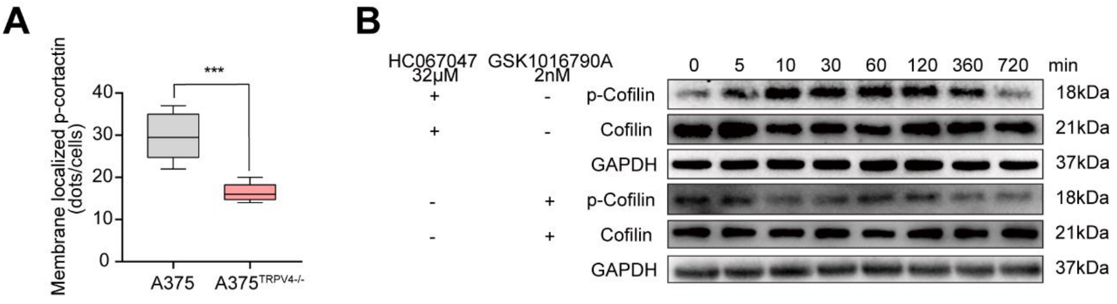

Supplementary Materials Figure S5.

The effect of HC067047 on the phosphorylation of cofilin. (A) Membrane localized P-Cortactin spot statistics in A375 and A375$^{TRPV4-/-}$ cells. (B) A375 cells and GSK1016970A were co-incubated for 5, 10, 30, 60, 120, 360 and 720 minutes respectively with or without HC-067047 preincubated for 30min. Data are expressed as means ± SD. ***P < 0.001.
